# Hyperemesis gravidarum and the dental patient

**DOI:** 10.1038/s41415-026-9534-9

**Published:** 2026-06-26

**Authors:** Katy Martin, Rose Legg

**Affiliations:** 403902518421192636527 Dublin Dental University Hospital, Lincoln Place, Dublin 2, D02 FE59, Ireland; 585056065445192010245 Community Dental Services CIC UK, England, United Kingdom

## Abstract

**Introduction** Hyperemesis gravidarum (HG) is a severe debilitating form of pregnancy sickness that affects around 3% of pregnancies annually. The annual cost to the National Health Service of nausea and vomiting in pregnancy has been estimated to be up to £62 million. Whilst the general focus of national guidance remains on the systemic management of HG, there is little recognition in the literature of the effects of hyperemesis and the oral cavity.

**Clinical features** The effects of prolonged vomiting can result in malnutrition, electrolyte imbalances and weight loss, in addition to the effects on the oral cavity including increased risk of caries, erosion, taste disturbances and xerostomia. Treatment options can include prescription of antiemetics, corticosteroids, enteral tube and parenteral feeding.

**Discussion** Maintaining good oral health during an HG pregnancy can be extremely challenging. As dental professionals, it is important to have a holistic understanding of HG, to be able to appropriately support patients and staff members who may be suffering with HG both pre-, peri- and post-partum.

**Conclusion** Hyperemesis gravidarum can be an isolating condition for pregnant people. It is essential that dental professionals are familiar with HG in order to adequately support staff members and patients who may be suffering.

## Key points

- Provides an overview of hyperemesis gravidarum and the relevance to the dental patient.
- Reviews the oral manifestations of hyperemesis gravidarum.
- Reviews common medications use in the management of hyperemesis gravidarum and the effects on the oral cavity.

## Introduction

Hyperemesis gravidarum (HG) is characterised in the Windsor definition as ‘a condition that starts early in pregnancy, before a gestational age of 16 weeks, and is characterised by severe nausea and/or vomiting, inability to eat and/or drink normally and strongly limits daily activities.'^1^ Whilst nine in ten pregnant people experience nausea and vomiting in pregnancy (NVP), HG is a severe debilitating form of pregnancy sickness that affects around 3% of pregnancies annually, and sits at the extreme end of the pregnancy sickness spectrum.^2^ The Windsor definition aims to move towards a subjective patient focused criteria with the hope to improve recognition and diagnosis of HG, shifting away from historical objective measures such as weight loss or electrolyte imbalance used in diagnosis.

NVP is one of the most common reasons for hospital admission among pregnant people, with typical stays of between three and four days.^3^ The severity of nausea can be compared to oncology patients treated with platinum-based chemotherapy which are known to be the most nauseating, with the same mechanism and pathways activated in HG patients.^4^

Historical understanding and treatment of people suffering from HG as a condition is laced in misconception, stigma and controversy. Charlotte Brontë is reported to have died from HG and the complications that can arise including refeeding syndrome.^5^ More recently, awareness of HG has been raised, with figureheads including the Princess of Wales reporting this condition during her pregnancies. However HG still remains a socially challenging and incapacitating condition.^6^ The annual cost to the NHS of nausea and vomiting in pregnancy has been estimated to be up to £62 million.^3^ Research and funding for vital medications for HG sufferers has been raised in UK Parliament as recently as July 2025, with the devastating consequences of treatment deprivation highlighted, including maternal suicide.^7^

Despite gradually increasing recognition of HG, there is a distinct lack of recognition of NVP and HG and the effect of these conditions on the oral cavity within the dental literature.

## Clinical features of hyperemesis gravidarum

The major mechanism of NVP and HG is deemed to be related to hypersensitivity to the vomiting hormone, growth differentiation factor 15 (GDF15).^3^ GDF15 is produced when genes in the placenta are activated, which in turn activates the vomiting centre within the brain, resulting in nausea and vomiting in animal models.^8^ Higher circulating levels of GDF15 are found in HG patients, those using anti-emetics and patients with continued second trimester vomiting.^1^ In spite of these genetic markers, HG remains a clinical diagnosis, with the side effects and complications of HG summarised in Table 1.^1^^,^^2^ The effects of prolonged vomiting can result in malnutrition, electrolyte imbalances and weight loss, in addition to the effects on the oral cavity. It is important to recognise the varying presentations of HG, with a no ‘one size fits all' list of clinical features, signs and symptoms.

**Table 1:** Side effects and complications which may present in people with hyperemesis gravidarum^3^^,^^9^

**Side effects and complications of hyperemesis gravidarum**		
Severe nausea	Hyponatraemia	Higher anxiety disorder*
Severe vomiting	Hypokalaemia	Higher depression scale*
Dehydration	Hypovolemia	Increased risk low birth weight
Inability to eat and/or drink normally	Metabolic hypochloraemic alkalosis	Increase risk of preterm birth
Limit on daily activities	Ketonuria	Increased risk venous thromboembolism
Taste aversions	Low blood pressure	Termination for medical reasons
Weight loss	Thyroid/renal/hepatic dysfunction	Side effects of medications
Headaches	Thyrotoxicosis	
Hypersalivation	Wernicke's encephalopathy	
*when compared to non hyperemesis gravidarum patients		

In addition to the physical difficulties arising with HG, there are significant psychological and emotional challenges that arise not only pre-partum but can continue post-partum. Studies have demonstrated statistically significant higher depression scale and anxiety disorder scale scores in people with HG. HG patients are also at an increased risk of developing post-traumatic stress disorder and post-natal depression.^9^ Maternal suicide ideation has also been observed in those with increased sickness severity, poor functional status and poor perception of quality of care.^10^ Maternal suicide has been reported as the second most common cause of direct maternal death in the UK, which is particularly pertinent given that risk factors include social isolation and pregnancy complications, both of which are frequently observed in people with HG.^10^^,^^11^ Referral to mental health services to provide people with an individualised emotional and psychological support plan is imperative.

## Treatment of hyperemesis gravidarum

The management of HG is dependent on the severity of the condition. The ‘pregnancy-unique quantification of emesis‘ (PUQE) is an objective and validated index of nausea and vomiting which can be used to classify the severity of NVP and HG.^3^ The severity of symptoms defined by the PUQE score or other validated measures dictate treatment. Treatment options can include prescription of antiemetics, corticosteroids, enteral tube and parenteral feeding. Correction of electrolyte or metabolic disturbances should also be considered.^3^

People may require hospitalisation in severe cases where symptoms cannot be appropriately managed in an outpatient setting. Treatment may include intravenous or parenteral fluids, vitamins and antiemetics. Thiamine supplementation should be given to all people admitted with prolonged vomiting to prevent Wernicke's encephalopathy.^3^ Patients should only be discharged from hospital once they are able to tolerate adequate oral nutrition, hydration and antiemetic therapies.^3^ Those who have nausea and vomiting but are not dehydrated can be cared for in the community through a combination of oral antiemetics and rehydration, dietary advice, psychological support and reassurance and validation of symptoms.

Specific consideration needs to be given to the increased risk of venous thromboembolism in people with HG. People admitted with HG should be offered thromboprophylaxis with low-molecular-weight heparin, which is particularly important given the clinically significant increase in the risk of venous thromboembolism in this patient group.^3^

A summary of the first-, second- and third-line medications used in the UK, recommended in the Royal College of Obstetricians and Gynaecologist Greentop Guidelines 2024 to treat NVP and HG can be found in Table 2. It is important that dentists have an awareness of the potential side effects and drug interactions seen with these medications. Of note all first line anti-emetics listed are antihistamines.

**Table 2:** Common medications used in the management of hyperemesis gravidarum^3^^,^^17^

**Drug name**	**Side effects relevant to dentists**	**Potential drug interactions relevant to dentists**
**First line**		
Doxylamine succinate and pyridoxine hydrochloride (Xonvea)	Dry mouth	Amitriptyline Codeine Diazepam Dihydrocodeine Gabapentin Midazolam Nitrous oxide Pregabalin Remimazolam Temazepam
Cyclizine	Dry mouth, dry throat, increased gastric reflux, leukopenia	Amitriptyline Codeine Diazepam Dihydrocodeine Gabapentin Lamotrigine Midazolam Remimazolam
Prochlorperazine (Stematil and Buccastem)	Oral disorders, nasal congestion, dry mouth	Amitriptyline Celecoxib Codeine Diazepam Etoricoxib Gabapentin Ibuprofen, Lamotrigine Midazolam Naproxen Nitrous oxide Remimazolam
**Second line**		
Metoclopramide hydrochloride	Dry mouth	Anaesthetic drugs including atracurium and rocuronium
Ondansetron	Dry mouth	Carbamazepine Erythromycin Tramadol
**Third line**		
Steroids (intravenous hydrocortisone followed by oral prednisolone)	Adrenal suppression, osteonecrosis, leukocytosis, increased infection risk, impaired healing	Aspirin Carbamazepine Erythromycin Etoricoxib Fluconazole Ibuprofen Naproxen

Whilst all therapeutic measures should be offered to people suffering with HG, termination of a wanted pregnancy is sometimes an unavoidable last resort and may be considered as part of a multidisciplinary plan.^3^ Documentation of therapeutic failure and seeking of a psychiatric opinion should be completed in cases where HG is the reason for termination, with people offered counselling before and after a decision of pregnancy termination is made.^3^ Sometimes HG (or the treatment of HG) may lead to life-threatening illness which can unfortunately result in termination of pregnancy to be seen as the only option.^3^

## Effects of hyperemesis gravidarum on the oral cavity

Whilst the general focus of national guidance remains on the systemic management of HG, there is little recognition in the literature of the effects of hyperemesis and the oral cavity. As dental professionals, it is important to have a holistic understanding of HG, to be able to appropriately support patients and staff members who may be suffering with HG both pre-, peri- and post-partum.

For patients with HG, nutrition and dietary intake will understandably alter. Studies have shown that pregnant people have a higher prevalence of dental caries, irrespective of HG, due to dietary changes, increased acidity in the oral cavity and reduction in saliva production.^12^ Flavoured fluids (often with sugar additives) and specific categories of foods including sweet or salty can be better tolerated, with the emphasis on eating foods patients can tolerate rather than those of high nutritional value.^13^ Patients may also be prescribed high calorie (and often high sugar content) nutritional supplementation alongside their normal diet. The move towards a cariogenic diet can mean that a previously ‘low caries risk' patient moves into a ‘high caries risk' category, and therefore their dental management and recall will need to be adjusted accordingly. Due to the nature of HG, patients may not present for routine dental care and instead only in acute clinical situations. It is important to maintain a non-judgemental and supportive approach, particularly given that studies have demonstrated the ‘stigma' attached to HG which can act as a barrier for people seeking healthcare.^14^ People with HG are more likely to suffer with dental erosion, due to intrinsic factors of gastric acid reaching the oral cavity as a result of prolonged vomiting.^15^ This can contribute to dental wear which can lead to dental issues post-partum.

For patients with HG, steroids can be trialled in cases where all other measures have failed.^3^ Duration of steroid therapy is dependent on therapeutic response, and a low maintenance dose may need to be considered until the gestational age at which HG will have typically resolved is reached.^3^ Complications of steroid use include adrenal insufficiency, risk of gestational diabetes and increased infection risk, which includes cervicofacial infections. In early pregnancy, steroid use has been reported to an increase the risk of cleft lip and/or palate, but this suggestion must be interpreted with caution given that these statistics may be due to chance rather than causation.^3^ It is important to remember that patients with HG may also have nutritional deficiencies, including folate. Folic acid is recommended to be taken in the first 12 weeks of pregnancy to reduce the prevalence of cleft lip and palate, alongside supporting brain and spinal cord development.^16^ Prolonged hyperemesis may prevent adequate intake and absorption of folic acid, which in turn could lead to developmental complications.

It is important to regard the side-effects to each medication outlined in Table 2,^3^^,^^17^ All first- and second-line medications listed have the potential side effect of xerostomia, which this is likely to greatly impact dental and oral health of the HG patient. In addition, it is likely HG patients are taking a combination of medication and this polypharmacy further increases their risk of xerostomia and hyposalivation.

Whilst one of the main side-effects of the medications used to manage HG is hyposalivation, people suffering with HG are also more likely to suffer with ptyalism.

Ptyalism can be highly distressing and is characterised by excess salivation and difficulty swallowing saliva, which can result in increased stimulation of the retching reflex.^18^ Not only can ptyalism be associated with embarrassment, anxiety and negative social stigma, sufferers may also present with dysgeusia, enlargement of salivary glands and erythematous tongues.^18^ The aetiology of ptyalism in pregnancy is unknown, but a strong bidirectional relationship has been identified between ptyalism gravidarum and HG, with one study reporting that 26% of people with nausea and vomiting complained of ptyalism in the first trimester.^18^^,^^19^

Unsuprisingly, HG can leave people with dental issues, causing embarrassment and hesitancy to seek dental treatment post-partum. One study demonstrated that 85% of participants reported that brushing or flossing ‘always' or ‘often' induced nausea and/or vomiting during HG pregnancy, with 97% reporting difficulty brushing or flossing at least ‘sometimes' during their pregnancy.^20^ Because of this, oral hygiene routines can be drastically altered during HG pregnancy, with 67% brushing teeth less than once a day during pregnancy.^20^

## Discussion

It is important to recognise that dental professionals may be exposed to HG not only through patient relationships, but through colleagues and other staff members. In 2024, 93% (72,983) of dental care professional registrants with the General Dental Council were women, with 53% of dentists registered as female.^21^ Given the stigma often attached to HG, it is important that employers are approachable and supportive. Workplace adjustments may include flexible working hours (to allow appointments to be planned around symptoms), closer access to a bathroom, and reduced workload (including shorter days) where appropriate.^22^

In addition to the physiological and physical effects of HG, patients may often be left with oral healthcare issues. The careful use of language is essential when supporting patients and staff with HG. NVP is often labelled as ‘morning sickness' which is felt by sufferers to be inaccurate and trivialise the condition.^3^ Ginger foodstuffs have been shown to have adverse and unpleasant effects for over half of people surveyed, and recommendations of such foods by healthcare professionals to patients with severe HG has been shown to damage clinician-patient relationships and erode trust in the healthcare professional.^3^^,^^23^ Patients have reported healthcare professionals to be dismissive and unsympathetic when seeking treatment for NVP and HG, and are often dissatisfied with communication during their appointments.^3^ Whilst dentists are unlikely to be the first point of call for people with HG, it is important that any clinical contact (e.g., in an acute dental emergency) remains a positive experience.

### Oral health recommendations for hyperemesis gravidarum patients

Maintaining good oral health during an HG pregnancy can be extremely challenging, with dietary changes, drug induced xerostomia, salivary changes and acid reflux. Simple measures to support patients should be utilised:^20^^,^^24^^,^^25^^,^^26^

- Brushing teeth twice daily – once at night and one other time of day. If brushing in mornings is challenging, timing of brushing can be altered to suit symptoms. Do not brush teeth within one hour of vomiting
- Use of SLS-free or flavour-free toothpaste - avoidance of strong tastes which may stimulate vomiting
- Use of stannous-fluoride containing toothpaste/mouthwashes may provide additional erosion protection
- Rinse mouth with water or alcohol-free mouthwash after vomiting
- Use of soft toothbrush/toothbrush with a smaller head to avoid activating gag reflex and risk of further vomiting
- Interdental plaque control before toothbrushing
- Encourage adoption of good dietary practice in line with the Eatwell Guide - be aware that dietary habits may alter during HG pregnancies to facilitate adequate calorie intake
- Avoid smoking in pregnancy.

### Further support

The following resources can be accessed by patients and staff members who may benefit:

- Pregnancy Sickness Support www.pregnancysicknesssupport.org.uk
- Hyperemesis Education and Research (HER) Foundation www.hyperemesis.org
- Hyperemesis Ireland www.hyperemesis.ie

## Conclusion

Hyperemesis gravidarum can be an isolating and debilitating condition for pregnant people. It is essential that dental professionals are familiar with HG in order to adequately support staff members and patients who may be suffering. The effects of HG, including those affecting the oral cavity, can continue post-partum. Further research is required to support the management of HG.
